# A meta-analysis of the activity, stability, and mutational characteristics of temperature-adapted enzymes

**DOI:** 10.1042/BSR20210336

**Published:** 2021-04-30

**Authors:** Stewart Gault, Peter M. Higgins, Charles S. Cockell, Kaitlyn Gillies

**Affiliations:** 1 UK Centre for Astrobiology, SUPA School of Physics and Astronomy, University of Edinburgh, James Clerk Maxwell Building, Peter Guthrie Tait Road, Edinburgh EH9 3FD, U.K.; 2 Institute for Astronomy, University of Edinburgh, Royal Observatory, Blackford Hill, Edinburgh EH9 3HJ, U.K.

**Keywords:** enzyme activity, enzymology, extremophiles, mutation, protein stability

## Abstract

Understanding the characteristics that define temperature-adapted enzymes has been a major goal of extremophile enzymology in recent decades. In the present study, we explore these characteristics by comparing psychrophilic, mesophilic, and thermophilic enzymes. Through a meta-analysis of existing data, we show that psychrophilic enzymes exhibit a significantly larger gap (T_g_) between their optimum and melting temperatures compared with mesophilic and thermophilic enzymes. These results suggest that T_g_ may be a useful indicator as to whether an enzyme is psychrophilic or not and that models of psychrophilic enzyme catalysis need to account for this gap. Additionally, by using predictive protein stability software, HoTMuSiC and PoPMuSiC, we show that the deleterious nature of amino acid substitutions to protein stability increases from psychrophiles to thermophiles. How this ultimately affects the mutational tolerance and evolutionary rate of temperature adapted organisms is currently unknown.

## Introduction

Extremophiles on Earth have become adapted to both high and low ‘extreme’ environmental temperatures. In the process of evolving to survive in such environments, they have had to adapt their biomolecular machinery to function at extreme environmental temperatures [1–3]. As enzymes are the major facilitators of biological reactions, they represent an important window through which temperature adaptation of organisms can be understood. Temperature-adapted enzymes exhibit adaptations to both their activity and stability. Psychrophilic enzymes, those adapted to low temperature environments, exhibit greater activity at low temperatures compared with mesophilic and thermophilic enzymes [4]. Thermophilic enzymes on the other hand are adapted to be both active and stable at extremely high environmental temperatures, even upwards of 100°C [5–7]. These adaptations are achieved through specific changes to an enzyme’s amino acid composition [8–13], secondary structure [14], and the number and type of intramolecular bonds present in the enzyme [15–19]. In this study, thermophilic (from environments of ∼55–60°C) and hyperthermophilic (from environments >80°C) enzymes are grouped together.

Many studies of temperature-adapted enzymes focus on what may be considered the main physical characteristics of an enzyme: its optimum temperature (T_opt_) and its melting temperature (T_m_). Unsurprisingly, it is observed that psychrophilic enzymes exhibit a lower T_opt_ and T_m_ than their mesophilic and thermophilic homologues. However, it was also observed that some psychrophilic enzymes exhibited a T_opt_ that was far from their T_m_ [20]. Here, we term this difference between T_opt_ and T_m_ as an enzyme’s ‘temperature gap’ (T_g_). It was initially suggested that this gap was due to the active site of psychrophilic enzymes being more thermolabile than the rest of the protein in order to have sufficient flexibility to achieve catalysis at low environmental temperatures [4,20,21]. However, alternative hypotheses have been proposed to account for this, such as the equilibrium model [22], macromolecular rate theory [23–25], and the loss of temperature-sensitive enzyme–substrate interactions [26]. However, as most studies focus on one type of enzyme across a small sample of species, it is difficult to understand how representative this phenomenon is across many enzyme types. Therefore, the first aim of the present study is to determine whether a large T_g_ can be characterised as a general feature of psychrophilic enzymes and to what extent we also see this phenomenon in mesophilic and thermophilic enzymes.

Another suggested characteristic of temperature-adapted organisms is that thermophiles exhibit particularly low mutational tolerance [27,28]. It has been suggested that the high temperatures of a thermophile’s environment make it particularly constrained by temperature-sensitive mutations. However, it has also been suggested that microbial communities actually evolve faster in extreme environments [29], seemingly in contrast with the predictions made by Drake [27]. This raises the question as to whether mutations themselves have a greater effect on thermophilic enzyme stability, or do thermophiles simply live closer to their proteome’s thermodynamic edge of stability than do mesophiles or psychrophiles? Thus, the second aim of the present study was to determine whether protein mutation software, PoPMuSiC [30] and HoTMuSiC [31], predicts a difference in effect to an enzyme’s Gibbs free energy of folding (ΔΔG_f_) or melting temperature (ΔT_m_) upon mutation among psychrophiles, mesophiles, and thermophiles.

In the present study, it is shown through meta-analysis that the T_opt_ and T_m_ of an enzyme increases from psychrophiles to thermophiles, as is expected. It is also shown that, while most enzymes exhibit a T_g_, the T_g_ of psychrophilic enzymes is significantly larger than that of both mesophilic and thermophilic enzymes and in certain cases T_g_ provides the best indication of whether an enzyme is psychrophilic or not. Additionally we show that the average amino acid substitution is more deleterious to thermophilic enzyme stability compared with psychrophilic enzymes, with a general increase in the deleterious effect from psychrophiles through to thermophiles. Owing to the small absolute predicted differences between the stability parameters for the temperature-adapted enzymes, it is unknown how this would affect the mutational tolerance of thermophiles compared with mesophiles and psychrophiles over evolutionary timescales.

## Methods

### Dataset construction

Two datasets were created for the present study. Dataset 1 contains the T_opt_ and T_m_ data for homologous temperature-adapted enzymes from psychrophiles, mesophiles, and thermophiles which were included following a literature search of published data. Dataset 1 also contains the calculated T_g_. T_g_ is defined here as the temperature gap between an enzyme’s T_m_, and its T_opt_ and is calculated from the following equation:

$$T_{g}=T_{m}-T_{opt}$$

Dataset 2 contains the Protein Data Bank (PDB) IDs of homologous temperature-adapted enzymes from psychrophiles, mesophiles, and thermophiles which were found following a literature search or from searching through the PDB itself.

Each dataset had certain criteria which had to be met before data were entered into the dataset. For dataset 1, only wildtype enzymes were included. This meant that variants generated through random/targeted mutagenesis were excluded. This means that the data obtained for the studied enzymes result from their natural evolutionary history, whereas generated variants may have contained alterations which are not represented or permissible in the natural environment and as such may have affected the results. For an individual enzyme, the T_m_ and T_opt_ values were only taken from separate publications if it was clear that both studies were using the same enzyme from the same source organism. Reports of T_50_ values were excluded as they primarily reflect the kinetic stability of an enzyme rather than the global stability which is inferred from T_m_ measurements. Reports in which an enzyme’s T_m_ was lower than its T_opt_ were excluded. Such reports were rare. Furthermore an enzyme was only included in dataset 1 if both T_m_ and T_opt_ could be obtained, otherwise T_g_ could not be calculated. This has bearing for the thermophilic results as there were instances of thermophilic enzymes exhibiting high T_opt_ values, however the T_m_ values were experimentally unobtainable in the respective studies. These restrictions on data mean that the results presented here may represent a lower estimate of the mean T_m_, T_opt_, and T_g_ of thermophilic enzymes.

Dataset 2 had similar entry requirements, such as only natural enzymes were included, and generated variants were excluded. As the mutational software used in the present study is structure based, a PDB ID was required for entry into dataset 2. Enzymes were taken as psychrophilic, mesophilic, and thermophilic based on how the source literature characterised them.

### Predicting the effect of mutations to protein stability

In order to predict the effect of mutations on the stability of temperature-adapted enzymes, two pieces of software were used, HoTMuSiC and PoPMuSiC [30,31] (available at https://soft.dezyme.com/). Both pieces of software require a PDB ID as input. HoTMuSiC predicts the effect of a mutation to a protein’s melting temperature (ΔT_m_), therefore a positive value is interpreted as stabilising and a negative value is destabilising. PoPMuSiC predicts the effect of a mutation to a protein’s ΔΔG_f_ and so a negative value is stabilising, and a positive value is destabilising. For data analysis, the mean effect of mutations to the respective proteins was recorded. Together the two pieces of software report on different, but complementary parts of a protein’s temperature stability curve.

### Statistics

Statistical analysis was performed on GraphPad Prism. The results were analysed for statistically significant differences using one-way ANOVAs followed by post-hoc Tukey’s multiple comparisons tests. If the group variances were found to be significantly different using a Bartlett’s test, then a Welch’s ANOVA was employed instead, followed by post-hoc Dunnett’s T3 multiple comparisons tests. This was implemented for the T_g_ and ΔT_m_ data. The ANOVA results and post-hoc test results are provided as supplementary information.

## Results

### Enzyme activity and stability

**Figure 1:**
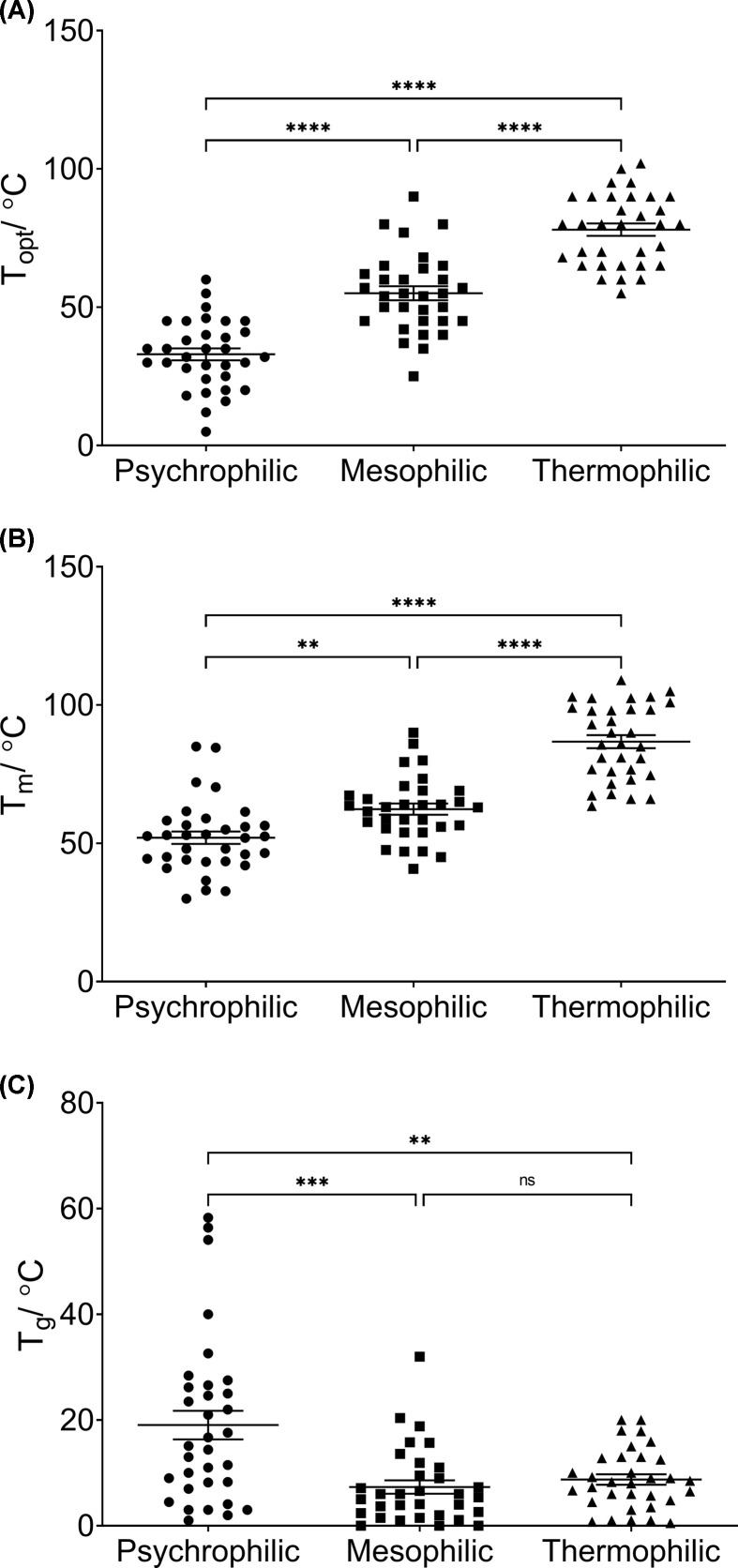
The activity and stability parameters of temperature-adapted enzymes Panel (**A**) represents the optimum temperature for enzyme activity (T_opt_), while (**B**) shows the melting temperatures (T_m_) of the individual enzymes. Panel (**C**) shows the temperature gap between T_opt_ and T_m_, denoted as T_g_. The individual data points for psychrophiles are represented by circles, mesophiles by squares, and the thermophiles by triangles. All data points are plotted with the mean ± the SEM. * represent the statistical significance results from Tukey’s multiple comparisons tests for panels (A,B) and Dunnett’s T3 multiple comparisons tests for panel (C) (** = *P*<0.01, *** = *P*<0.001, **** = *P*<0.0001, ns = not significant).

The first hypothesis tested in the present study is to what extent can T_opt_, T_m_, and T_g_ be described as defining characteristics of temperature-adapted enzymes. Figure 1 shows the T_opt_ (A), T_m_ (B), and T_g_ (C) of enzymes from temperature-adapted organisms. The results displayed in Figure 1A show that the T_opt_ of an enzyme increases with increasing environmental temperatures and that the T_opt_ values were significantly different in pairwise comparisons (*P*-values, psychrophile-mesophile = 4.2 × 10^−9^, psychrophile-thermophile = 5 × 10^−10^, mesophile-thermophile = 1.4 × 10^−9^). The mean T_opt_ values (±SEM) for psychrophilic, mesophilic, and thermophilic enzymes are 32.97 (±2.16), 55.03 (±2.52), and 78.03 (±2.25)°C respectively.

Similarly, Figure 1B shows that the T_m_ of an enzyme increases from psychrophiles to thermophiles and that T_m_ values were significantly different in pairwise comparisons (*P*-values, psychrophile-mesophile = 0.004, psychrophile-thermophile = 5 × 10^−10^, mesophile-thermophile = 5 × 10^−10^). The mean T_m_ values for psychrophilic, mesophilic, and thermophilic enzymes are 55.02 (±2.25), 62.37 (±2.02), and 86.77 (±2.38)°C respectively.

The statistically significant difference between the means of both T_opt_ and T_m_ for all three groups of organisms suggests that, on average, T_opt_ and T_m_ can be described as defining characteristics of an enzyme from organisms adapted to different temperature conditions. Namely, that psychrophiles exhibit the lowest T_opt_ and T_m_ as they inhabit the lowest temperature environments, while the opposite is true for the thermophiles with the mesophiles exhibiting intermediate values.

Figure 1C shows that while all enzymes exhibited a T_g_, it is only statistically significantly different when comparing psychrophilic enzymes to mesophilic or thermophilic enzymes (*P*-values = 0.000896 and 0.00276 respectively). There is no statistical difference between the T_g_ of mesophilic enzymes and thermophilic enzymes (*P*-value = 0.765462). The mean T_g_ for psychrophiles is 19.05 (±2.71)°C whereas for mesophiles and thermophiles it is 7.34 (±1.26) and 8.74 (±0.99)°C, respectively. So while most enzymes exhibit a T_g_, it is significantly greater in psychrophilic enzymes. These results suggest that a large T_g_ may be considered as an indicative characteristic of psychrophilic enzymes in general, analogous to their canonical characteristics of a lower T_opt_ and T_m_.

### Effect of mutations

**Figure 2:**
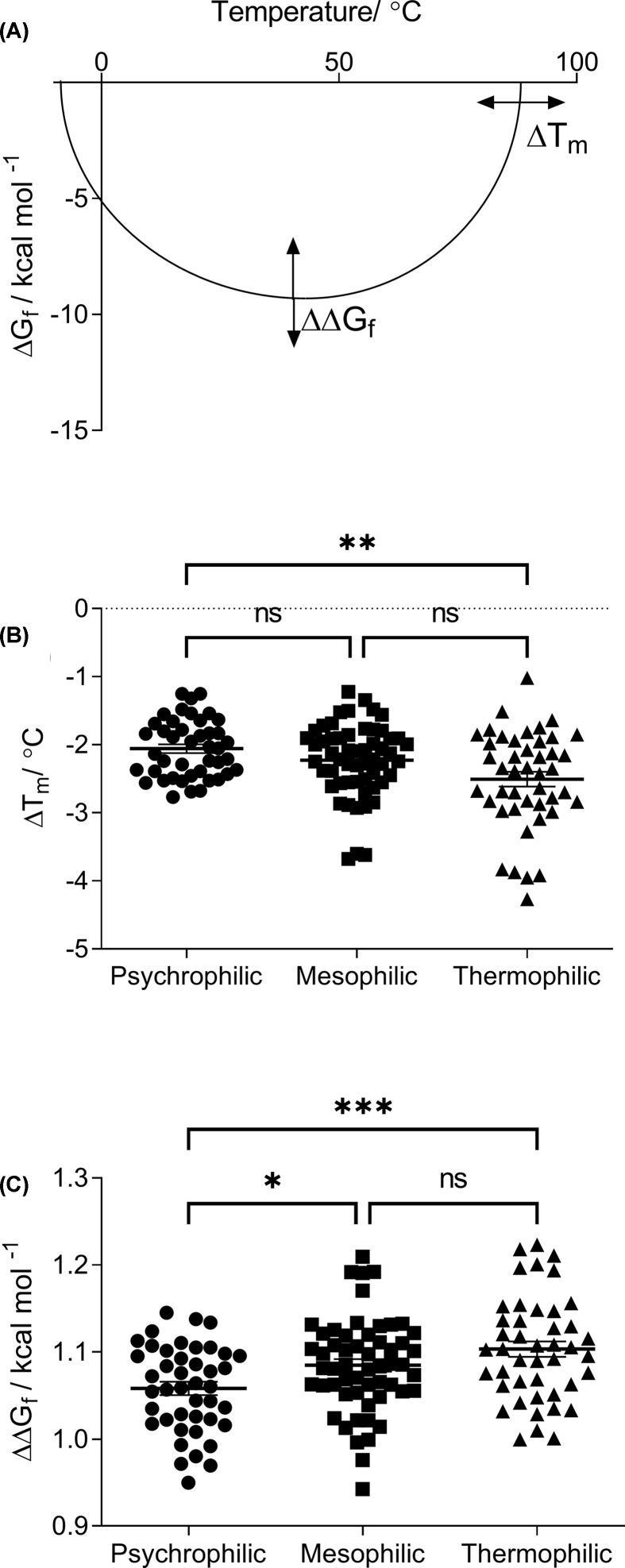
The effects of mutations to temperature-adapted enzymes Panel (**A**) shows a representative protein stability curve expressed as its Gibbs free energy of folding (ΔG_f_) across temperature. The stability curve exhibits two melting points where it crosses the x-axis, and a peak of stability where the curve has its most negative y value. Horizontal and vertical arrows represent the changes to protein stability predicted by HoTMuSiC and PoPMuSiC respectively (ΔT_m_ and ΔΔG_f_). Panel (**B**) shows the ΔT_m_ predicted by HoTMuSiC to enzymes from psychrophiles, mesophiles, and thermophiles as a result of single amino acid mutations. Panel (**C**) shows the ΔΔG_f_ predicted by PoPMuSiC to enzymes from psychrophiles, mesophiles, and thermophiles. All data points are plotted with the mean ± the SEM. * represent the statistical significance results from Dunnett’s T3 multiple comparisons tests for panel (B) and Tukey’s multiple comparisons tests for panel (C) (* = *P*<0.05, ** = *P*<0.01, *** = *P*<0.001, ns = not significant).

The second hypothesis tested in the present study was that there was a difference in the effect of a mutation (specifically amino acid substitutions) to an enzyme’s ΔΔG_f_ or melting temperature (ΔT_m_) among psychrophiles, mesophiles, and thermophiles. Figure 2A shows a representative protein stability curve which could be produced with results from differential scanning calorimetry with the Gibbs free energy of folding on the y-axis and temperature on the x-axis. A protein’s stability curve shows a region of peak stability where ΔG_f_ is most negative, and also exhibits two melting points where the curve intersects the x-axis. On Figure 2A, the horizontal and vertical arrows represent the changes to a protein’s melting temperature and Gibbs free energy of folding predicted by HoTMuSiC and PoPMuSiC respectively.

Figure 2B shows the predicted ΔT_m_ to enzymes from psychrophiles, mesophiles, and thermophiles upon mutation by HoTMuSiC. The average ΔT_m_ was −2.06, −2.23, and −2.51°C for psychrophiles, mesophiles, and thermophiles respectively. The results suggest that the average reduction in the melting temperature of an enzyme upon mutation increases from psychrophiles to thermophiles, which agrees with previous literature [32]. The only Dunnett’s T3 multiple comparisons test to produce a statistically significant result was between the psychrophiles and the thermophiles (*P*-value = 0.0019). The difference among the three categories is small in terms of absolute numbers, but as percentages they suggest that the average mutation to a thermophilic enzyme is ∼10–25% more destabilising than those to their mesophilic and psychrophilic counterparts. To what extent such differences would have an effect over evolutionary timescales is currently unknown.

Figure 2C shows the predicted ΔΔG_f_ to enzymes from psychrophiles, mesophiles and thermophiles upon mutation by PoPMuSiC. The average ΔΔG_f_ was 1.058, 1.085, and 1.103 kcal.mol^−1^ for psychrophiles, mesophiles, and thermophiles respectively. Similar to the ΔT_m_ results, the average ΔΔG_f_ upon mutation increases from psychrophiles through to the thermophiles. Post-hoc Tukey’s multiple comparisons tests showed statistically significant differences between psychrophiles-mesophiles and psychrophiles-thermophiles with *P*- values of 0.0451 and 0.000459, respectively.

From these results it is demonstrated that the average mutation to an enzyme not only lowers the melting temperature, but also reduces the thermodynamic stability, thus constricting the global folded phase space. Furthermore, it is shown that mutations are more deleterious to thermophilic enzymes than they are to mesophilic or psychrophilic enzymes.

## Discussion

In this meta-analysis, we have collated and presented data which further expand our understanding of the defining characteristics of temperature-adapted enzymes. It was shown that the T_opt_ and T_m_ of enzymes increased with increasing environmental temperatures. In contrast, it was shown that an enzyme’s T_g_, the gap between the optimum and melting temperature of an enzyme, is significantly larger in psychrophiles, and is in fact a defining characteristic of psychrophilic enzymes that could allow for the prediction of enzymatic psychrophilicity. Additionally it was shown that the average amino acid mutation is predicted to be more destabilising to thermophilic enzymes than it is to mesophilic or psychrophilic enzymes.

Our data allow for several important observations. There is a considerable overlap in the T_m_ values for psychrophiles and mesophiles, suggesting that increased psychrophilic enzyme activity at lower temperatures has not necessarily come at a cost to overall protein stability. This suggests that global protein stability is not a major constraint on psychrophilic enzyme adaptation and evolution. Conversely, thermophilic enzyme stability is more clearly an adaptive feature as seen from the larger difference between the thermophilic and mesophilic T_m_ means.

The results show that not all psychrophilic enzymes necessarily have psychrophilic characteristics. This is perhaps best exemplified by one of the enzymes included in our dataset, the thermostable psychrophilic glutathione reductase from an Arctic *Sphingomonas* with a T_opt_ and T_m_ of 60 and 84.6°C respectively [33], values typically associated with thermophilic enzymes. In this case, the large T_g_ value of 24.6°C is the best predictive indicator that this enzyme came from a psychrophilic organism. Additionally, few psychrophilic enzymes exhibit T_opt_ values which would be considered similar to the expected environmental temperature of a psychrophile.

It should also be noted that the thermophile T_m_ and T_opt_ (and consequently T_g_) values represent a lower estimate of their true population. This is due to exclusion of studies which did not report both the T_m_ and T_opt_. This largely results from the limitations of circular dichroism apparatus and differential scanning calorimeters used in such studies, which prevent the measurement of high T_m_ values. It raises a question as to whether there are thermophilic enzymes which are so thermostable that they resist melting until their carbon backbone begins to physically dissociate. The sample size of thermophilic enzymes was further reduced due to the propensity to report T_50_ measurements in the literature. This is understandable due to the considerable biotechnological interest in thermophilic enzymes [1], where their kinetic stability at elevated temperatures is of more interest than the temperature at which global unfolding occurs.

These data also raise the question of the correlation between enzyme type and the size of T_g_. Evidence for a correlation was seen with the luciferase enzymes included in our dataset. They exhibit high T_g_ values in both psychrophiles and mesophiles. Our dataset contained four luciferase enzymes. The three psychrophilic luciferase T_g_ values were 56.4, 58.3, and 54.1°C with a mesophilic firefly luciferase exhibiting a T_g_ of 15.8°C. Of additional interest is the observation that all three psychrophilic luciferases were more thermostable than the mesophilic firefly luciferase, by as much as 31°C.

While our results show that a large T_g_ is a defining characteristic of psychrophilic enzymes, they cannot elucidate the precise source of this phenomenon. We can however discuss the consequences of each hypothesis with regards to our analysis. Multiple explanations have been proposed to explain this observation such as, active site unfolding [4,20,21], an equilibrium model [22], macromolecular rate theory [23–25], and the loss of specific temperature-sensitive enzyme–substrate interactions [26]. The initial explanation that the active site of α-amylase from the psychrophile *Pseudoalteromonas haloplanktis* is particularly thermolabile [21] possesses strong explanatory power and fits with observations that increased active site flexibility and dynamics are key to achieving greater enzymatic activity at low environmental temperatures [34–37]. Within the framework of this hypothesis, our results would suggest that, as a population, psychrophilic enzymes possess significantly more thermolabile active sites than do mesophiles or thermophiles compared with the stability of the whole enzymes. An equilibrium model interpretation of the data would suggest that psychrophilic enzymes reach the equilibrium temperature (T_eq_), the point at which half the enzyme is active, much before they reach their T_m_. This would suggest that the ratio of active to inactive enzyme forms (E_act_/E_inact_) is particularly temperature sensitive in psychrophiles and therefore results in a larger T_g_. The loss of temperature-sensitive enzyme–substrate interactions proposed by Sočan et al. [26] is largely a molecular level interpretation of the equilibrium model as they propose a ‘dead-end model’ where an inactive enzyme forms with increasing temperature. This would suggest that the interactions between substrates and psychrophilic enzymes is significantly weaker than those of mesophilic and thermophilic enzymes and therefore is the source of the large T_g_ in psychrophilic enzymes. Macromolecular rate theory would predict that the change in heat capacity of activation (ΔC*_p_*^‡^) is significantly lower in psychrophilic enzymes compared with mesophilic and thermophilic enzymes. This would cause a larger T_g_ in psychrophiles due to the increasing curvature of the temperature-dependent activity profile as ΔC*_p_*^‡^ is lowered. No single hypothesis may explain the T_g_ phenomenon and diverse hypotheses may be applicable to different enzymes. It will require precise measurements on the molecular level to determine the true origin of T_g_ for each enzyme.

The lower T_g_ values for mesophilic and thermophilic enzymes may be useful for validating ancestrally reconstructed enzymes. Ancestral reconstruction tends to produce more thermostable enzymes [38,39], however there is a concern that this may be an artifact due to biases in the reconstruction process [40]. Therefore based on our meta-analysis, if these ancestral enzymes were indeed more thermophilic, then one should not expect to find that T_g_ increases significantly when constructing an ancestral enzyme from the modern day mesophilic form.

The mutational data presented here are in strong agreement with the well-established observation that mutations are on average destabilising. The ΔT_m_ values reported here are less destabilising than those presented in previous literature [32] which ranged from approx. −1.3 to −5°C, with thermophilic proteins predicted to experience more destabilising mutations. This may be due to the focus on enzymes in the present study, which may produce more stabilising mutations than the average non-enzymatic protein. This could be explained by the fact that the active site of an enzyme generally contributes little to stability, therefore mutating it tends to introduce stabilising interactions [41–44] or have more neutral effects. Our data does however point towards an increasing trend in this deleterious nature with increasing environmental temperatures. Therefore, studies regarding the trajectories and timescales of enzyme evolution may require varied weighting of mutational effects depending on the thermophilicity of the enzymes in question.

The observation that mutations are more deleterious to thermophilic enzymes agrees with the hypothesis put forward by Drake [27]. If there is a tight coupling between a thermophile’s environmental temperature and its enzymes’ temperature stabilities, then a difference in ΔT_m_ of 0.5°C may be sufficient to make the average mutation particularly potent against thermophile survivability. So while thermophilic proteins may be more tolerant to mutations at ∼30°C compared with their mesophilic counterparts [45], the coupling of environmental temperature and T_m_ would produce the phenomenon of lower mutational tolerance *in situ*. In contrast with Drake [27], Li et al. [29] have reported that microbial communities evolve faster in extreme environments. Drake reported that the d_N_/d_s_ (the non-synonymous/synonymous mutation ratio) for thermophiles was lower for thermophiles compared with mesophiles, 0.09 versus 0.14 respectively, suggesting thermophiles tolerate less mutation. However, Li et al. report that communities of thermophiles from hot springs have a higher d_N_/d_s_ than communities from the surface ocean, freshwater or soil (d_N_/d_s_ values of 0.126, 0.061, 0.087, and 0.087 respectively). Li et al. also reported higher relative evolutionary rates (rERs) for thermophilic communities compared to freshwater and soil communities. It is hard to directly compare the two studies though, as Drake [27] considered other mutation types such as chain terminations and indel mutations. On the other hand, Drake [27] examined two species of thermophiles, so it is difficult to extrapolate those results to all thermophiles, whereas Li et al. have reported data at the community level, making their work potentially more representative of thermophiles as a class of organism. The experimental determination of whether psychrophiles and mesophiles can tolerate higher mutational loads than thermophiles, while critical for answering this question, is limited by the long time-course required to culture and grow such organisms.

## Conclusion

The aim of the present study was to further explore the characteristics of temperature-adapted enzymes. It was shown, in strong agreement with theory, that the T_opt_ and T_m_ increases with an organism’s environmental temperature. It was also shown that a large T_g_ is a defining characteristic of psychrophilic enzymes and in certain cases is a better predictor of psychrophilicity than either T_opt_ or T_m_. The average effect of an amino acid mutation to temperature-adapted enzymes was also explored. It was found that the average ΔT_m_ and ΔΔG_f_ becomes more deleterious, with increasing environmental temperature. The difference in deleterious effect was small and the effect of this over evolutionary timescales is unknown.

## Supplementary Material

Supplementary Data Files

## Data Availability

The source data for all results are provided as Supplementary Data. Dataset 1 contains the T_m_, T_opt_ and T_g_ values for each enzyme, their source organism, and their literature source. Dataset 2 contains the PDB IDs of all enzymes used in the mutation results with their average ΔT_m_ and ΔΔG_f_. Both datasets contain a summary table. The ANOVA results and post-hoc test results for each analysis are also provided as a supplementary data file.

## Open Access

Open access for this article was enabled by the participation of University of Edinburgh in an all-inclusive *Read & Publish* pilot with Portland Press and the Biochemical Society under a transformative agreement with JISC.

## Abbreviations

PDB: Protein Data Bank
T_g_: temperature gap
T_m_: melting temperature
T_opt_: optimum temperature
ΔΔG_f_: Gibbs free energy of folding
